# The role of integrins in glioma biology and anti-glioma therapies

**DOI:** 10.1186/2193-1801-4-S1-L12

**Published:** 2015-06-12

**Authors:** Patrick Roth

**Affiliations:** University of Zurich, Switzerland

**Keywords:** Glioma, integrin, TGF-beta

Integrins are a group of molecules expressed by various cells including glioma cells and endothelial cells within the tumor. There are 18 known alpha and beta integrin subunits which form a heterodimer. Integrins regulate different cellular processes such as proliferation, adhesion, motility and survival as shown in numerous preclinical models. Furthermore, integrins control the activity of the transforming growth factor (TGF)-beta pathway and are involved in the process of angiogenesis which is indispensable for continued tumor growth. Because of the high expression levels of some integrins on glioma cells and their numerous functions, inhibition of integrin signaling has been considered a promising strategy for the treatment of glioma patients. Besides blocking antibodies which are currently under clinical investigation in other cancer entities, the integrin inhibitor cilengitide has been tested within several trials in glioblastoma patients over the last years. Cilengitide is a cyclic RDG peptide which targets integrins alphvbeta3 and alphavbeta5. Based on the results of smaller, initial trials suggesting an activity of cilengitide against glioblastoma, 2 larger trials were subsequently performed. However, both trials, which combined temozolomide-based chemoradiation with cilengitide failed to demonstrate an improved outcome with the addition of cilengitide. Ongoing translational analyses suggest that integrin levels in the tumor tissue are neither prognostic nor predict response to cilengitide. While the clinical development of cilengitide has been stopped, integrin inhibition with more effective agents may still be a promising approach in clinical neurooncology.

